# Lung function decline after mountain ultra-marathon is accompanied by reduced inspiratory pressure but not by detectable changes in CC16

**DOI:** 10.1007/s00421-026-06349-9

**Published:** 2026-07-21

**Authors:** Antoine Raberin, Bastien Krumm, Tom Citherlet, Guia Tagliapietra, Tadej Debevec, Vincent Pialoux, Grégoire P. Millet

**Affiliations:** 1 Institute of Sport Sciences, Faculty of Biology and Medicine, University of Lausanne, Lausanne, CH-1015 Switzerland; 2 Faculty of Sports, University of Ljubljana, Ljubljana, Slovenia; 3 Department of Automation, Biocybernetics and Robotics, Jožef Stefan Institute, Ljubljana, Slovenia; 4 Atherosclerosis, Thrombosis and Physical Activity Team, Interuniversity Laboratory of Human Movement Biology UR7424, Université Claude Bernard Lyon 1, Univ Lyon, Lyon, France

**Keywords:** Pulmonary system, Ultra-endurance, Ultra-trail, Altitude, Environmental physiology

## Abstract

**Purpose:**

Mountain ultra-marathons (MUM) impose multiple stressors on pulmonary function inducing risk of airway epithelial damage. This study aimed to assess the magnitude of pulmonary function changes and the associated risks of airway damage induced by such a race.

**Methods:**

Thirteen volunteers completed the MUM (330 km, 24,000 m elevation gain). Pulmonary function was assessed using standardized procedures before (pre-) and within 1 h after (post-) the race. Assessments included forced inhalation and exhalation maneuvers, as well as maximal inspiratory pressure (MIP) measurements. Venous blood samples were collected for the analysis of plasma club cell secretory protein 16 (CC16). Blood volume was determined using the carbon monoxide rebreathing method.

**Results:**

From pre- to post-, forced vital capacity (FVC) (-7.5%, *p* = 0.001), forced expiratory volume in 1 s (FEV_1_) (-12.2%, *p* = 0.002) and peak expiratory flow (-10.4%, *p* = 0.016) were reduced. MIP decreased by 14.5% (104 ± 29 vs. 90 ± 26 cmH₂O, *p* = 0.005). CC16 levels remained unchanged, even after normalization for plasma volume changes (*p* = 0.929). Slope ratio and β-angle of the maximal expiratory flow-volume curve changes were not significant (*p* = 0.101 and *p* = 0.055, respectively). Significant relationships showed that greater decreases in FVC (*r* = − 0.60, *p* = 0.026) and FEV₁ (*r* = − 0.62, *p* = 0.018) were associated with faster MUM completion times.

**Conclusion:**

The acute negative impact of MUM on pulmonary function has been confirmed. The extent of this impairment appears to be proportional to race duration. Biomarker analysis suggests that airway epithelial integrity remains preserved. The reduced pulmonary function could be due to respiratory muscle fatigue.

## Introduction

In recent years, participation in ultra-endurance events has significantly increased, particularly in running events (Scheer 2019). Ultra-endurance running is typically defined as any race longer than the marathon distance (42.195 km) or exceeding six hours for timed endurance events (Scheer et al. 2020).

Mountain ultra-marathon (MUM) imposes specific challenges encountered by participants (e.g., prolonged exercise for very long period, high altitude, extreme distance and elevation, changes in ambient temperature, backpack), that may particularly affect pulmonary function.

First, at altitude, reduced barometric pressure lowers air density and the partial pressure of inspired oxygen. Additionally, lower ambient temperatures at altitude result in the inhalation of cooler air (Cogo 2011). In response to the hypoxic environment, ventilation increases to maintain arterial oxygenation, a process known as the hypoxic ventilatory response (HVR) (Teppema and Dahan 2010). Additionally, hypoxic pulmonary vasoconstriction (HPV) redirects blood flow from hypoxic to well-ventilated alveoli to optimize ventilation-perfusion matching (Dunham-Snary et al. 2017). However, when hypoxia is generalized across the lungs, HPV increases pulmonary arterial pressure and may contribute to subclinical or pulmonary edema (Dunham-Snary et al. 2017; Swenson 2020). Such pulmonary edema may occur during a MUM, as evidenced by the reported decrease in lung diffusing capacity and increase in lung comet tails post MUM (Stewart et al. 2024).

Secondly, the prolonged duration of the race exposes participants to a wide range of environmental conditions, including repeated transitions between high and low altitudes, fluctuations in temperature and humidity between day and night, and variable weather conditions throughout the course. At altitude, ventilation during exercise is augmented relative to normoxia at a given submaximal workload, partly reflecting the contribution of the hypoxic ventilatory response to overall ventilatory drive (Constantini et al. 2021). This sustained hyperpnea may contribute to airway epithelial stress and injury during mountain ultra-marathon. Indeed, among the mechanisms known to compromise airway integrity, prolonged hyperpnea and inhalation of cold, dry air are the primary contributors (Atchley and Smith 2020; Karamaoun et al. 2022).

Finally, the physiological strain induced by the mandatory equipment carried in a backpack during the race should not be neglected. Research has shown a progressive reduction in forced vital capacity (FVC) with increasing backpack loads during short uphill walking bouts (Dominelli et al. 2012). Moreover, prolonged thoracic loading has been shown to exacerbate respiratory muscle fatigue after 60 min of walking (Faghy and Brown 2014). While no specific data exist on the impact of prolonged carriage of lighter loads during multi-day events like MUM, this aspect could play a role in the pulmonary function decline associated with ultra-endurance activities.

Taken together, the characteristics of MUM (e.g., prolonged duration, altitude exposure, and environmental conditions) impose substantial physiological demands on the pulmonary system. Notably, a 13% prevalence of exercise-induced bronchoconstriction has been reported in a large cohort (1212) of ultra-marathon runners (Hoffman and Krishnan 2014).

While the alteration of lung function during MUM remains a primary respiratory concern (Tiller et al. 2020), the body of evidence remains comparatively small and characterized by inconsistent findings (Tiller 2019). For instance, some studies reported no change in FVC or maximal inspiratory pressure (MIP) (Warren et al. 1989; Wüthrich et al. 2015; Martínez-Navarro et al. 2020), while others observed significant reductions (Mahler and Loke 1981; Martínez-Navarro et al. 2020), likely due to differences in race distance and conditions. Notably, spirometry and maximal voluntary ventilation were measured during a previous edition of the same MUM (Vernillo et al. 2015) and indicated decreases in FVC, forced expiratory volume in 1 s (FEV₁), and maximal voluntary ventilation, suggesting that changes in lung elastic properties, interstitial pulmonary edema, and/or respiratory muscle fatigue underly the observed impairments. However, none of these studies investigated biological evidence of any alteration of the airway integrity in this type of event. Club cell secretory protein 16 (CC16), a mediator released by airway epithelial cells in response to airway dehydration and cooling caused by high minute ventilation, has been used to assess airway epithelial integrity in the context of endurance sports (Kippelen and Anderson 2012; Pourmanaf et al. 2023), but to the best of our knowledge, has never been measured following a MUM, leaving airway epithelial integrity after such events unknown.

The purpose of this study was to confirm the decline in pulmonary function following a MUM and to investigate whether it is accompanied by changes in respiratory muscle strength and/or airway epithelial damage, in order to explore the potential mechanism and the clinical relevance of this alteration. We hypothesized that MUM induces airway epithelial damage (assessed via circulating CC16) and impairs pulmonary function (assessed via spirometry) through airway obstruction and respiratory muscle fatigue (assessed via MIP).

## Materials and methods

### Participants

Twenty-four participants (5 women and 19 men) volunteered to take part in the study. Among them, 13 (4 women and 9 men) successfully completed the MUM.This corresponds to a 65% finishing rate, consistent with previous studies on the same event (Saugy et al. 2013; Vernillo et al. 2015).

Eligibility criteria included being free from any known diseases, non-smokers, having no history of asthma, and not taking any medication during the race.

The study was conducted in accordance with the Declaration of Helsinki and was approved by the local ethics committee of the *Azienda Regionale Sanitaria USL della Valle d’Aosta* (06/05/2021.0038781.I). All participants gave their written informed consent before participation.

### Experimental design

The study was conducted on *the Tor des Géants 2023*. Considered as one of the most challenging MUM, the *Tor des Géants* is a single-stage race that covers 330 km with 24,000 m of elevation gain. The current records are 64 h 43 min 57 s for men and 79 h 10 min 40 s for women, while the maximum time allowed to complete the race is 150 h. The average altitude of the race is 1,944 m, with 168 km taking place at elevations above 2000 m. During the race, ambient temperature and relative humidity varied substantially along the course. Based on available meteorological data from the Aosta valley, participants were exposed to temperatures ranging from approximately 2–5 °C at the highest altitude sections (> 3,000 m) during nighttime to approximately 18–22 °C at lower altitude during the day, with relative humidity ranging from ~ 48–100% (daytime) to ~ 60–100% (nighttime). Participants were initially tested 24 to 48 h before the race and within 1 h after arrival. All measurements were conducted in the same temperature-controlled indoor room (Courmayeur, Italy, ~ 1224 m).

### Pulmonary function test

Participants were seated comfortably with the trunk at 90°. A portable spirometer (Pony FX, Cosmed, Rome, Italy) and an electronic pressure transducer (KH2, PowerBreathe International Ltd., Warwickshire, UK) were used. Devices were calibrated according to manufacturer instructions.

FVC and MIP maneuvers were performed according to American Thoracic Society/European Respiratory Society guidelines (American Thoracic Society/European Respiratory Society 2002; Graham et al. 2019). Predicted values and z-scores were computed using the Global Lung Function Initiative (GLI) multi-ethnic reference equations (Quanjer et al. 2012). A single trained investigator conducted all tests and interpreted the data to ensure consistency and validity.

The following variables were derived from maximal flow-volume curves (MFVC): FVC, FEV_1_, peak expiratory flow (PEF), and forced expiratory flow between 25 and 75% of FVC (FEF_25%−75%_). Participants were required to complete at least three acceptable MFVC trials lasting ≥ 6 s (with two largest FVC and FEV_1_ within 150 mL), with adequate recovery between attempts. Among the acceptable maneuvers, the trial with the highest sum of FVC and FEV_1_ was selected for analysis. MIP was measured from residual volume according to ATS/ERS guidelines, using a minimum of three technically acceptable maneuvers with less than 10% variability between the highest values, and the greatest of these reproducible efforts was recorded as MIP (American Thoracic Society/European Respiratory Society 2002).

To quantify the curvature of the MEFVC, the slope ratio (SR) and β-angle were calculated. The SR determination has been previously described (Dominelli et al. 2016). Briefly, both a tangent line and a chord line were determined between 20% and 80% of the FVC. The tangent at each point of interest was calculated using expiratory flow and volume values 100 mL before and after that point. The chord line was defined as the straight line connecting the point of interest to residual volume. The SR was computed as the ratio of the slope of the tangent line to that of the chord line. The individual SR was obtained by averaging SR values across the 20–80% FVC range.

The β-angle was calculated as:

$$\:{\upbeta\:}=\:180-{tan}^{-1}\left(\frac{PEF\:-\:{MEF}_{50\%}}{0.5\:\times\:\:FVC}\right)+\:{tan}^{-1}\left(\frac{{MEF}_{50\%}}{0.5\:\times\:\:FVC}\right)$$

were MEF50% is the maximum expiratory flow at 50% of FVC.

### Blood sampling and club cell secreted protein measurement

Venous blood samples (6 mL) were collected from the antecubital vein by a medical doctor while participants were seated. Samples were collected in BD Vacutainer EDTA-K2 tubes (Cat. No. 368856, USA), centrifuged for 10 min at 3,500 rpm. Plasma was aliquoted and immediately stored at − 80 °C.

Plasma concentrations of CC16 were quantified using ELISA (MyBiosource, San Diego, CA, USA) and read on a TECAN Infinite 2000 plate reader (Männedorf, Switzerland), in accordance with manufacturer instructions.

To account for potential post-race hemodilution, absolute values of CC16 were also adjusted using plasma volume measurements (data and procedure previously published (Krumm et al. 2024). Briefly, plasma volume was calculated from total hemoglobin mass, hemoglobin concentration, and hematocrit (Siebenmann et al. 2017). Total hemoglobin mass was measured using an automated CO-rebreathing system (OpCo, Detalo Instruments, Copenhagen, Denmark) as previously described (Siebenmann et al. 2017). Participants inhaled a CO/O₂ gas mix for 6 min, followed by 4 min of ambient air. CO dose was 1 mL/kg (men) and 0.8 mL/kg (women). Capillary blood samples were analyzed in triplicate for carboxyhemoglobin and hemoglobin concentration (ABL800, Radiometer, Copenhagen, Denmark). Residual CO was measured (Monoxor Plus, Bacharach, New Kensington, PA, USA) to determine the exact bolus. Hematocrit was assessed via microcentrifugation (SERVOspin Smart Micro, Servoprax GmbH, Germany).

### Statistical analysis

A statistical power analysis was performed to determine the appropriate sample size for this study. The a priori sample size was based on previous results (Vernillo et al. 2015), which reported a significant decrease in FEV_1_ after a similar MUM with a moderate to strong effect size (0.68) (Vernillo et al. 2015). Using a significance level of 0.05 and a desired power of 0.80, the analysis indicated that a sample size of 15 finishers would be required to detect a pre- to post-race decrease in FEV_1_ using paired t-test. To account for the high dropout rate typically observed in such events, 24 participants were recruited to reach the desired sample size.

Data were first assessed for normality using Q–Q plots and skewness inspection. To compared pre- vs. post-race values, a paired Student t-test was used when data followed a normal distribution. For non-normally distributed variables, Wilcoxon signed-rank test was applied. Pearson correlation coefficients were calculated to measure strength of the linear relationship between pre- and post- race changes and the time to complete the race (normally distributed). Effect size was calculated using Hedges g, appropriate for small sample size when data were normally distributed and rank-biserial effect size for non-normally distributed data. All statistical analyses were performed using IBM SPSS Statistics (Version 29.0, IBM, NY, USA). Data are reported as mean ± SD.

## Results

The finishers were 41.8 ± 7.0 years old, weighed 68.3 ± 9.7 kg, had a height of 174.7 ± 7.7 cm, and a BMI of 22.3 ± 2.2 kg·m⁻². On average, participants completed the race in 123 ± 24 h and performed the pulmonary function test 38.41 ± 8.32 min after their arrival. One participant out of 13 exhibited abnormal pulmonary function prior to the race (Z-score = − 1.75 (FVC) and − 3.33 (FEV_1_)). All the significant findings remained the same when this participant was excluded from the analysis.

**Fig. 1:**
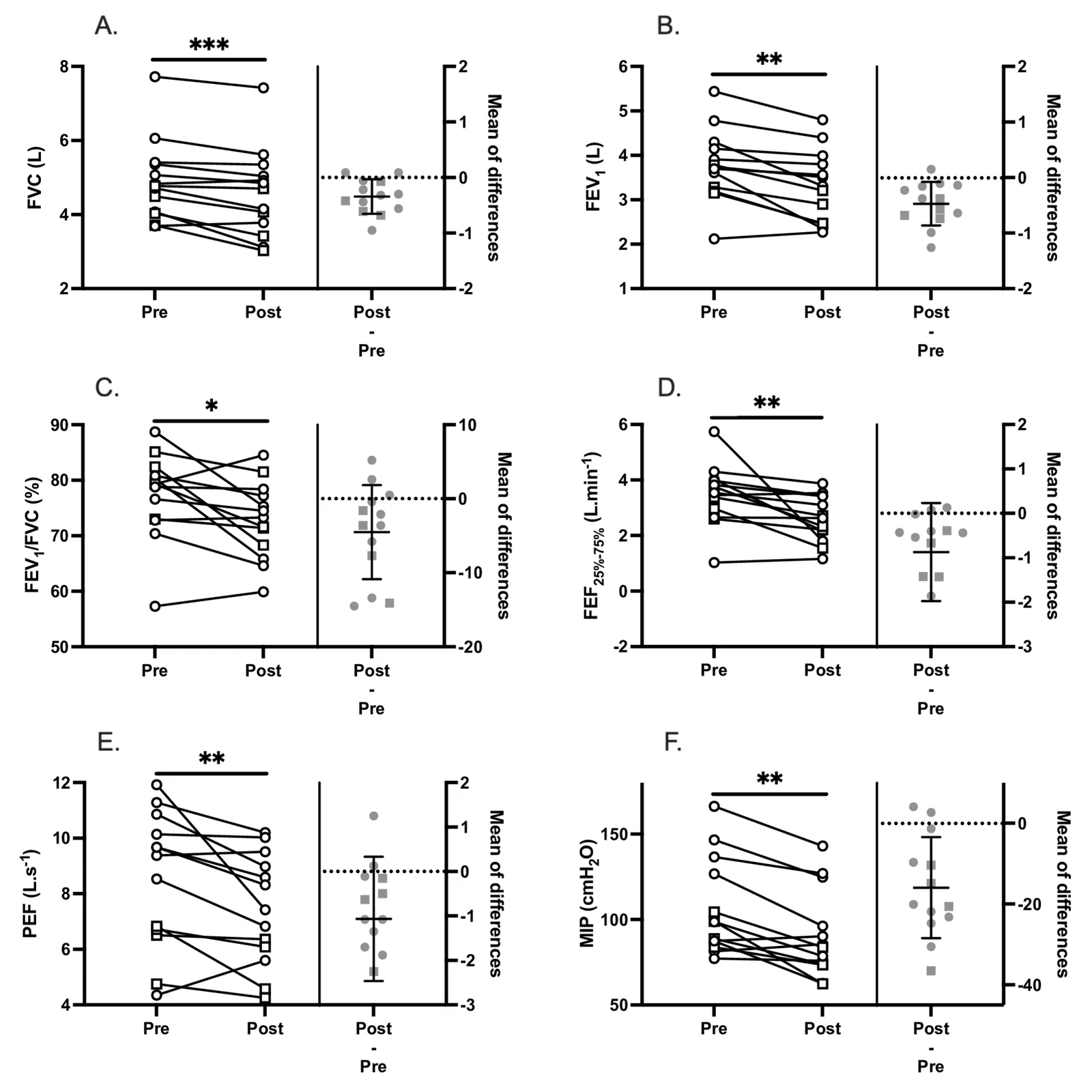
Spirometry parameters (**A**, **B**, **C**, **D**, **E**) and maximal inspiratory pressure (MIP) (**F**) changes in male (n=9) and female (n=4) finishers pre- and within 1-hour post-race. Circles are men, squares are women. FVC: forced vital capacity. FEV1: forced expiratory volume in 1 second. FEF25%-75%: forced expiratory flow between 25–75% of FVC. PEF: peak expiratory flow. MIP: Maximal inspiratory pressure. * p < 0.05, ** p < 0.01, *** p < 0.001

**Fig. 2:**
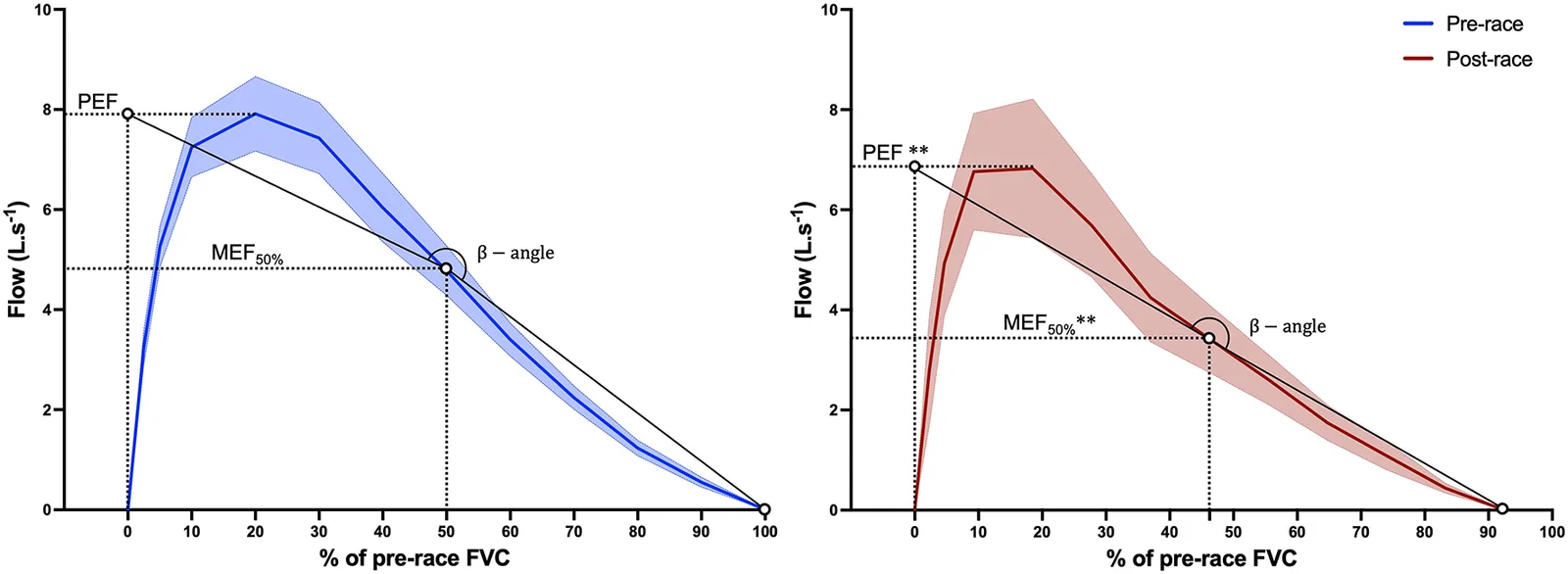
Mean maximal expiratory flow–volume curve pre- (blue) and post-race (red), expressed as percentages of 10% increments of pre-race forced vital capacity (FVC) in male (n=9) and female (n=4)finishers pre- and within 1-hour post-race. The shaded area represents the 95% confidence interval. PEF (peak expiratory flow) and MEF (maximum expiratory flow at 50% of forced vital capacity (FVC)) were significantly lower post-race (** p < 0.01). β-angle was over 180° in pre-race while it was over 180° post-race, but the difference did not reach statistical significance (p=0.055

From pre- to post-, spirometry indices showed significant reductions: FVC decreased by 7.5% (t = 4.10, *p* = 0.001, g = 1.06; Figs. 1A and 2), FEV_1_ by 12.2% (t = 4.84, *p* < 0.001, g = 1.26; Fig. 1B, FEV_1_/FVC ratio by 5.4% (W = 11.00, *p* = 0.016, *r* = 0.67; Fig. 1C), FEF_25%−75%_ by 20.6% (t = 3.29, *p* = 0.006, g = 1.16; Fig. 1D), and PEF by 10.4% (t = 2.80, *p* = 0.016, g = 1.50; Fig. 1E). MIP was reduced by 14.5% (W = 5.00, *p* = 0.005, *r* = 0.78; Fig. 1F). SR increased from 1.50 ± 0.23 to 2.02 ± 0.99, while the β-angle decreased from 188.3 ± 24.6° to 178.2 ± 19.5° (Fig.2); however, neither change reached statistical significance (SR: W = 69.00, *p* = 0.101, *r* = 0.45; β-angle: W = 18.00, *p* = 0.055, *r* = 0.53).

Percent predicted values for FVC, FEV_1_, FEV_1_/FVC, and FEF_25%−75%_ were significantly lower after the race (Table 1).

**Fig. 3:**
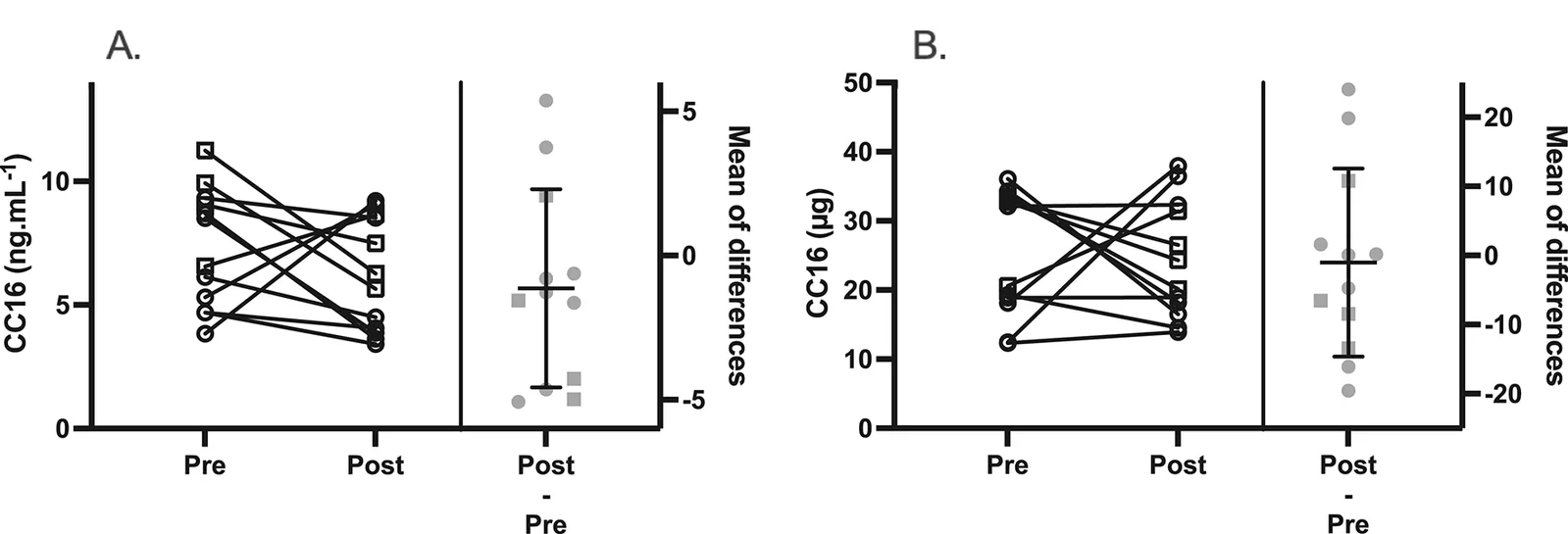
Changes in club cell secretory protein 16 (CC16) concentration (**A**) and absolute quantity (**B**) in male (n=8) and female (n=4) finishers pre- and within 1-hour post-race. Circles are men, squares are women

CC16 levels remained unchanged from pre- to post-race (t = 1.156, *p* = 0.270, g = 0.3; Fig. 3A), even after normalization for plasma volume changes (t = 0.091, *p* = 0.929, g = 0.02; Fig. 3B).

**Fig. 4:**
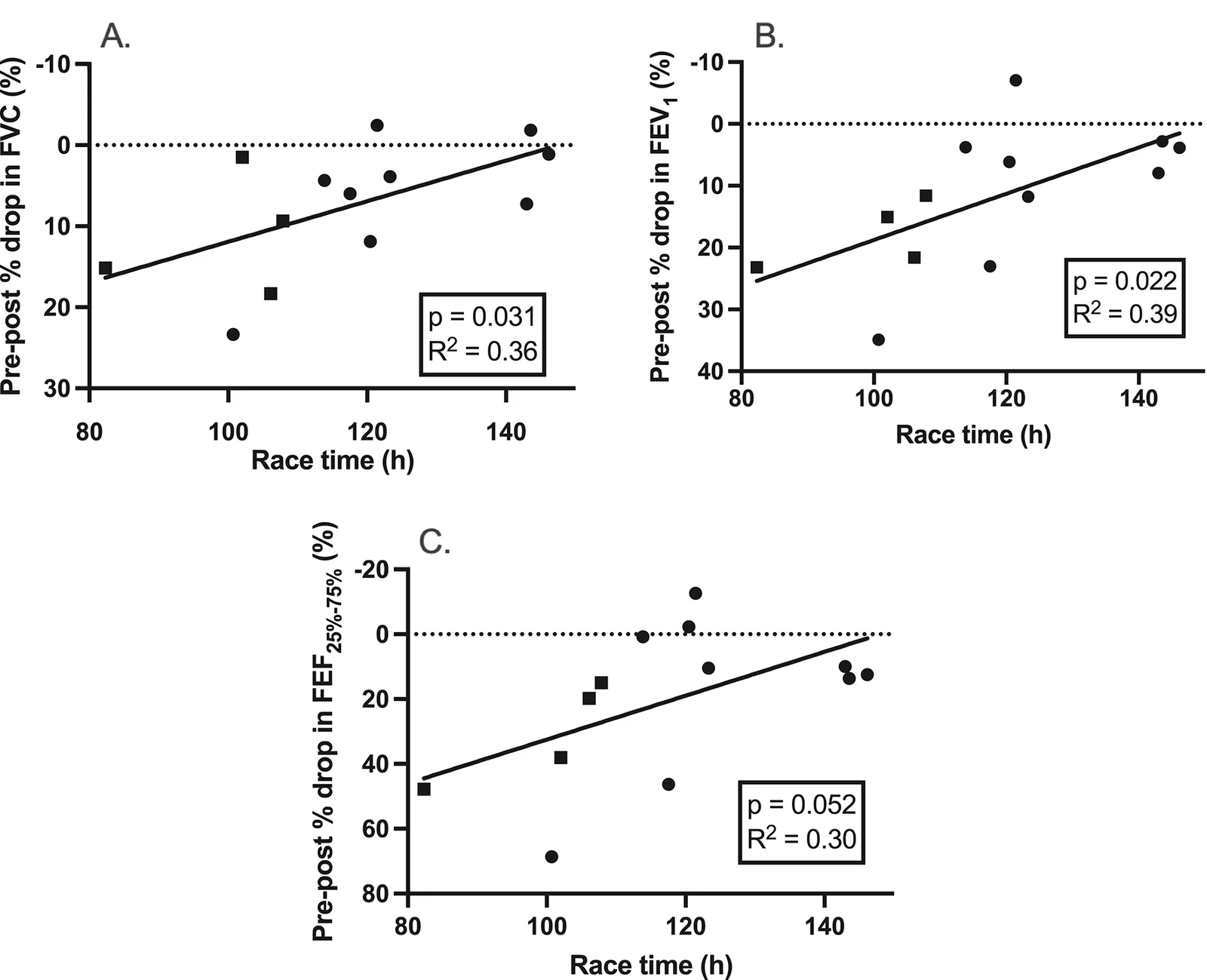
Relationship between race time and forced vital capacity (FVC) (**A**), forced expiratory volume in 1 second (FEV1) (**B**), and forced expiratory flow between 25–75% of FVC (FEF25%-75%) (**C**) in male (n=9) and female (n=4) finisher. Circles are men, squares are women

Significant negative correlations were observed between the pre-to-post-decreases in FVC (*r* = −0.60, *p* = 0.031) or in FEV_1_ (*r* = −0.62, *p* = 0.022) (Fig. 4A and B) and the race duration (i.e., faster runners showed higher pulmonary alteration). There was no other significant relationship with the race duration. Pre-to-post changes in MIP did not correlate with changes in spirometry indices. Exclusion of the participant with abnormal lung function prior the race slightly strengthened the association, however, the correlation remained non-significant (*r* = 0.53, *p* = 0.074).

Post-race, 2 participants (15.4%) exhibited a FVC value below lower limit of normal (LLN), 5 (38.5%) a FEV₁ value below the LLN, and 6 (46.2%) a FEF_25%−75%_ values below the LLN. Moreover, 7 of 13 participants (54%) met the criterion for exercise-induced bronchoconstriction (i.e., a reduction in FEV₁ >10%).

**Table 1:** Pre- to post-race changes in participants’ values of spirometry

Variable	Pre	Post	Test statistic	Effect size	*p*-value
FVC predicted (%)	102 ± 13	94 ± 16	t = 3.88	g = 1.00	0.001
FEV_1_ predicted (%)	99 ± 16	85 ± 15	W = 2.50	*r* = 0.83	0.003
FEV_1_/FVC predicted (%)	96 ± 10	90 ± 9	t = 3.07	g = 0.80	0.010
FEF_25%−75%_ predicted (%)	97 ± 31	70 ± 23	t = 3.24	g = 0.84	0.007

*FVC* forced vital capacity, *FEV*_*1*_ forced expiratory volume in 1 s, *FEF*_*25%−75%*_ forced expiratory flow between 25–75% of FVC, *PEF* peak expiratory flow, *MIP* maximal inspiratory pressure

## Discussion

This study investigated the alterations in pulmonary function observed during a MUM and their potential underlying mechanisms. The main findings of the present study were largely consistent with our hypotheses: pulmonary function was significantly impaired following the MUM, and this impairment was accompanied by a reduction in maximal inspiratory pressure, consistent with inspiratory muscle fatigue. However, contrary to our hypothesis regarding airway epithelial damage, CC16 concentrations did not increase from pre- to post-race, even after correction for the hemodilution known to occur during MUM (Krumm et al. 2024). This negative finding should be interpreted cautiously given the limitations inherent to this biomarker and study design, including the small sample size and the reliance on a single circulating marker.

Importantly, Vernillo et al. (2015) conducted their measurements during a previous edition of the same MUM, making the present study a partial replication, with the addition of MIP and CC16 measurement. Explicit replication in the same ecological context strengthens the robustness of the spirometric findings. Indeed, the magnitude of decline in FVC, FEV₁, and PEF was 7.5%, 12.2%, and 10.4%, respectively, compared to 9.5%, 9.7%, and 8.7% previously reported (Vernillo et al. 2015). Small differences may be explained by confounding factors such as participants pacing strategies, sex, hypoxic dose or weather conditions.

This observed decline in spirometry indices is consistent with a subset of the existing literature on ultra-marathon events, which so far has yielded inconclusive results. Reductions in FVC, FEV_1_, and PEF have been reported after races of 100 km (Mahler and Loke 1981) and 107 km (Martínez-Navarro et al. 2020). However, this is not a universal finding. Wüthrich et al. (2015) did not report any change in FVC, although FEV_1_ and PEF were reduced following a 110 km MUM (Wüthrich et al. 2015). More recently, no changes in FVC, FEV_1_, or PEF were observed in a ten-marathon stage race over 10 consecutive days (Tiller et al. 2019). Similar results were found when spirometry was performed every 3 h throughout a 24-hour ultra-marathon (Warren et al. 1989).

Although this cannot be directly verified, these discrepancies may be due to the prolonged altitude exposure during the present MUM (average altitude of the race of 1,944 m; 168 km at elevations above 2000 m; one peak > 3300 m). Indeed, alterations in pulmonary function have been reported when races expose participants to higher elevations (Wüthrich et al. 2015; Vernillo et al. 2015).

This impairment in lung function may be attributed to airway obstruction, as the FEV_1_/FVC ratio was also significantly reduced post-race. Prolonged hyperpnea during endurance exercise can result in dehydration and cooling of the airway mucosa (Atchley and Smith 2020). The subsequent release of inflammatory mediator may lead to vasoconstriction of airway vessels (Anderson and Daviskas 2000). Upon cessation of exercise, rewarming of the airways has been suggested to cause rebound hyperemia and vascular leakage, contributing to airway narrowing (McFadden et al. 1986). However, this later mechanism was not directly assessed in the present study and deserve further investigation. The marked reduction in FEF_25%–75%_ is consistent with involvement of the peripheral airways. In contrast to PEF, which is strongly effort-dependent, mid-expiratory flows are less influenced by initial expiratory effort (Mead et al. 1967). Consistent with this interpretation, the post-race flow–volume curves exhibited a more pronounced concave configuration. However, quantitative indices of curvilinearity did not reach statistical significance (SR: 1.50 ± 0.23 to 2.02 ± 0.99, *p* = 0.101; β-angle: 188.3 ± 24.6° to 178.2 ± 19.5°, *p* = 0.055). Although effect sizes were moderate, the lack of statistical significance likely reflects interindividual variability and the concomitant reduction in FVC, which may have compressed the expiratory volume range, thereby reducing the sensitivity of slope ratio and β-angle to detect subtle changes in curve concavity.

Interestingly, in our study, the magnitude of the decrease in FVC and FEV₁ was negatively correlated with race time. This suggests that race intensity, rather than cumulative duration alone, contributes to pulmonary function decline, potentially through higher ventilatory demands per unit time in faster runners. However, given the correlational nature of this finding and the small sample size, this interpretation should be regarded as exploratory. One could hypothesize that race intensity, alongside the duration of high-altitude exposure, is a key factor driving pulmonary function alterations during mountain ultra-marathons.

The 14.5% decrease in MIP observed in the present study is consistent with previous findings following ultra-marathon races conducted at altitude (Wüthrich et al. 2015; Martínez-Navarro et al. 2020; Martinez-Navarro et al. 2021), and contrasts with studies performed near sea level in which MIP was preserved (Warren et al. 1989; Ker and Schultz 1996; Tiller et al. 2019). Exposure to hypoxia, which is known to exacerbate respiratory muscle fatigue (Reinhard et al. 2023), may partly account for this discrepancy, and confirms the specific physiological demands of ultra-marathons performed in mountain environments (Millet et al. 2025).

Another factor that could have influenced the development of respiratory fatigue, specific to MUM, is the fact that participants carried a backpack. The prolonged carriage of an external load could impose additional strain on the respiratory muscles, but this is not consistently described across studies, even though it may contribute to fatigue development. Indeed, backpacks weighing 15 to 35 kg have been shown to reduce FVC (Dominelli et al. 2012) and increase respiratory muscle fatigue (Faghy and Brown 2014) after relatively short bouts of exercise (max 60 min) compared to MUM. Whether the prolonged carriage of a light load (~ 3–5 kg) over the duration of a MUM contributes meaningfully to respiratory muscle fatigue cannot be determined from the present data. To the best of our knowledge, the combination of carrying a light backpack over such a prolonged period has never been investigated. Therefore, the specific impact of this prolonged thoracic load carriage during MUM warrants further investigation.

Pre-to-post changes in MIP did not correlate with changes in FVC, FEV_1_, or PEF. However, this absence of a direct statistical association should not be interpreted as evidence against a contribution of respiratory muscle fatigue to the spirometric decline. MIP, measured from residual volume, reflects maximal static inspiratory force at low lung volume and does not capture the ability to generate rapid contractions and high flow during forced expiratory manoeuvres. A decrease in the rate of force development of the inspiratory muscles may therefore impair flow-dependent spirometric variables without being detected by static MIP measurements. This dissociation between MIP and spirometry has been previously reported in the ultra-marathon literature (Wüthrich et al. 2015).

In the absence of residual volume (RV) and total lung capacity (TLC) measurements, it is not possible to determine whether the observed FVC reduction reflects a decrease in TLC (consistent with inspiratory muscle fatigue and reduced ability to achieve full lung inflation), an increase in RV (consistent with expiratory flow limitation or air trapping), or a combination of both. Future studies incorporating body plethysmography or gas dilution techniques would provide a more comprehensive mechanistic interpretation of post-race spirometric changes.

This study also investigated alterations in epithelial airway integrity by measuring circulating levels of CC16. Prolonged hyperpnea during endurance exercise can lead to airway dehydration and, in some cases, cooling of the airways. These stressors stimulate epithelial cells to release inflammatory mediators (Daviskas et al. 1991). CC16 is an anti-inflammatory protein secreted by epithelial cells in the terminal bronchioles. When epithelial integrity is compromised, CC16 can diffuse into the bloodstream (Hermans et al. 1998). Therefore, elevated blood levels of CC16 are considered a biomarker of airway epithelial damage (Kippelen and Anderson 2012).

Previous studies have reported that increased ventilation during endurance exercise may cause epithelial damage and result in elevated CC16 concentrations (Bolger et al. 2011; Kippelen and Anderson 2012; Pourmanaf et al. 2023). This has been particularly observed following half marathons conducted in varying environmental conditions (at 19.3 °C, 20.5 °C, and 9.8 °C) (Chimenti et al. 2010), but to our knowledge has never been investigated during MUM.

In the present study, no significant change in CC16 concentration was observed from pre- to post-race. Importantly, MUM are known to induce a large hemodilution (Krumm et al. 2024), which can affect the interpretation of plasma and serum biomarker concentrations. To account for this, CC16 values were normalized for changes in plasma volume (Krumm et al. 2024). This correction supported the initial observation that CC16 levels remained unchanged post-race.

It is known that exercise intensity during such MUM remains relatively low, ranging from 52 to 65% of maximal oxygen uptake during uphill sections and even lower during downhill segments (Savoldelli et al. 2017). One may therefore speculate that the ventilation rate during the race did not exceed the individual-specific threshold necessary to induce epithelial damage. It has been suggested that such damage occurs only when this threshold is surpassed, resulting in airway dehydration and epithelial injury (Karamaoun et al. 2022). While this threshold may vary depending on exercise duration and environmental conditions, the ventilation rate during the present MUM was likely insufficient to disrupt epithelial integrity. Moreover, the intermittent pattern of the race, with participants alternating uphill and downhill sections and taking breaks to eat and sleep, may also have played a role (Combes et al. 2019).

However, average values should be interpreted with caution. As illustrated in Fig. 3, three participants exhibited marked increases in both relative and absolute CC16 concentrations, underscoring the importance of accounting for individual variability when assessing epithelial responses to extreme endurance exercise. However, as participants were assessed only once before and after a single race and no control condition was included, the extent to which the observed variability in CC16 responses reflects true individual differences versus normal biological or measurement variability in the context of a MUM remains uncertain.

## Perspective

The clinical relevance of these changes in pulmonary function may be questioned. Indeed, while the pre- to post-race decrease in spirometry outcomes are significant, LLN were not always crossed. Post-race, 2 participants exhibited FVC values below the lower LLN, 5 exhibited FEV_1_ values below the LLN, and 6 exhibited FEF_25%−75%_ values below the LLN. It should be noted that one participant already exhibited values below the LLN prior to the race, which is consistent with the pre-existing airflow obstruction identified in this individual.

If a reduction in FEV_1_ greater than 10% is used as a criterion for airway constriction (Kippelen et al. 2018), 7 out of 13 participants (i.e., 54%) meet this threshold, indicating significant airway narrowing.

In the context of a MUM, where inspiratory muscle fatigue is pronounced, the relevance of these clinical thresholds may be limited. Indeed, muscle fatigue may confound interpretations based solely on airway mechanics.

Moreover, previous studies investigating pulmonary function following marathons have demonstrated a partial recovery within 4 h and a complete return to baseline by 24 h post-race (Ross et al. 2008). It is therefore plausible that the alterations observed after such races are similarly transient, with spirometry indices normalizing as respiratory muscle fatigue resolves. Further studies are needed to characterize the pulmonary function recovery following MUM.

### Limitations

This study has several limitations. First, all pulmonary function tests used were volitional in nature and therefore subject to participant effort. However, standardized and replicable maneuvers were required to validate each test, which helps mitigate the influence of motivation. Additionally, although inspiratory muscle strength could have been assessed more objectively using nerve stimulation techniques, such methods are less practical in field conditions. In contrast, mouth-pressure maneuvers, while effort-dependent, are non-invasive, reproducible (Dimitriadis et al. 2011), and easier to implement in the field setting of a MUM.

It is important to note that the timing of post-race measurements is crucial and differed across studies. For instance, in the study by Ker and Schultz (1996) (Ker and Schultz 1996), assessments were conducted three days after arrival of the race, which may have blunted transient changes in respiratory muscle strength. Indeed, in marathon runners, partial and complete recovery of inspiratory muscle strength has been reported as early as 4 and 24 h post-race, respectively (Ross et al. 2008). In the present study, participants were tested as soon as possible after their arrival (38.41 ± 8.32 min). As the time course of lung function changes following ultra-endurance events has not been fully characterized, the lack of strict standardization in spirometry timing may introduced variability in the outcomes. This highlights the need for tighter post-race timing control and for the use of a broader range of biomarkers to improve understanding of the mechanisms underlying post-race functional changes.

Beyond respiratory muscle fatigue, additional mechanisms may have contributed to the transient post-race expiratory airflow limitation. Dehydration of the airway surface liquid during sustained hyperpnea increases its osmolarity. This hyperosmolar stimulus triggers the release of bronchoconstrictive mediators in the airway mucosa, a mechanism well established in the pathophysiology of exercise-induced bronchoconstriction (Kippelen and Anderson 2012). Whether this mechanism contributed to the post-race spirometric decline observed here remains to be determined, as neither airway surface liquid osmolarity nor bronchoconstrictive mediator concentrations were assessed in the present study.

The present study was not powered to detect changes in CC16, as the absence of prior data from mountain ultra-marathons precluded a CC16-specific sample size calculation during study design. Retrospective power estimates based on existing literature are possible but contextually limited: using the pooled effect size from a recent meta-analysis (g ≈ 0.54), a sample of 24 completers would be required (α = 0.05, power = 0.80). However, this meta-analysis included studies of ≤ 2 h duration at relatively high exercise intensities, which differ substantially from the present MUM (~ 123 h, predominantly at low-intensity). Given this unique context, no fully appropriate reference exists to quantify the underpowering precisely. The observed effect size (g = 0.30) should therefore be regarded as the first available estimate for this specific context and used to power future investigations.

Approximately 35% of recruited participants did not complete the race and were consequently not assessed. A survivor bias cannot be excluded: participants who withdrew may have included those with the greatest physiological strain, including potential airway epithelial injury (reflected in higher CC16) or more pronounced pulmonary function impairment. All findings should therefore be considered specific to race finishers and may not extend to non-finishers.

Finally, although sex-based differences in respiratory function during exercise and altitude exposure are well documented (Raberin et al. 2023), our sample size, despite considerable effort to include both sexes, was insufficient to allow for sex-specific comparisons.

## Conclusion

The suspected acute negative impact of mountain ultra-marathons on pulmonary function, with concomitant inspiratory muscle fatigue, has been confirmed. This impairment appears to be related to race duration, with faster athletes experiencing a greater decline. Furthermore, no group-level increase in the circulating airway epithelial injury marker CC16 was detected, though definitive conclusions regarding epithelial integrity cannot be drawn from a single biomarker. These findings suggest that, despite the extreme stress imposed on airways during mountain ultra-marathons, inspiratory muscle fatigue and potential airway obstruction, are primary contributors to the observed decline in pulmonary function.

## Data Availability

The data support the findings of this study are available from the corresponding author upon reasonable request.

## Declarations

The study was approved by the local ethical committee of the Azienda Regionale Sanitaria USL della Valle d’Aosta (06/05/2021.0038781.I).
